# Deconstructing Interocular Suppression: Attention and Divisive Normalization

**DOI:** 10.1371/journal.pcbi.1004510

**Published:** 2015-10-30

**Authors:** Hsin-Hung Li, Marisa Carrasco, David J. Heeger

**Affiliations:** 1 Department of Psychology, New York University, New York, New York, United States of America; 2 Center for Neural Science, New York University, New York, New York, United States of America; University of Tübingen and Max Planck Institute for Biologial Cybernetics, GERMANY

## Abstract

In interocular suppression, a suprathreshold monocular target can be rendered invisible by a salient competitor stimulus presented in the other eye. Despite decades of research on interocular suppression and related phenomena (e.g., binocular rivalry, flash suppression, continuous flash suppression), the neural processing underlying interocular suppression is still unknown. We developed and tested a computational model of interocular suppression. The model included two processes that contributed to the strength of interocular suppression: divisive normalization and attentional modulation. According to the model, the salient competitor induced a stimulus-driven attentional modulation selective for the location and orientation of the competitor, thereby increasing the gain of neural responses to the competitor and reducing the gain of neural responses to the target. Additional suppression was induced by divisive normalization in the model, similar to other forms of visual masking. To test the model, we conducted psychophysics experiments in which both the size and the eye-of-origin of the competitor were manipulated. For small and medium competitors, behavioral performance was consonant with a change in the response gain of neurons that responded to the target. But large competitors induced a contrast-gain change, even when the competitor was split between the two eyes. The model correctly predicted these results and outperformed an alternative model in which the attentional modulation was eye specific. We conclude that both stimulus-driven attention (selective for location and feature) and divisive normalization contribute to interocular suppression.

## Author Summary

In interocular suppression, a visible target presented in one eye can be rendered invisible by a competing image (the competitor) presented in the other eye. This phenomenon is a striking demonstration of the discrepancy between physical inputs to the visual system and perception, and it also allows neuroscientists to study how perceptual systems regulate competing information. Interocular suppression has been explained by mutually suppressive interactions (modeled by divisive normalization) between neurons that respond differentially to the two eyes. Attention, which selects relevant information in natural viewing condition, has also been found to play a role in interocular suppression. But the specific role of attentional modulation is still an open question. In this study, we proposed a computational model of interocular suppression integrating both attentional modulation and divisive normalization. By modeling the hypothetical neural responses and fitting the model to psychophysical data, we showed that interocular suppression involves an attentional modulation selective for the orientation of the competitor, and covering the spatial extent of the competitor. We conclude that both attention and divisive normalization contribute to interocular suppression, and that their impacts are distinguishable.

## Introduction

The perception of a brief target stimulus presented to one eye is suppressed by the simultaneous presentation of a dissimilar competitor stimulus to the other eye. This phenomenon, called interocular suppression, can be so strong that it renders the (otherwise easily visible) target invisible [1–3, reviewed in 4]. It has been hypothesized that normalization [5, 6] contributes to the neural processing underlying interocular suppression [7–10], in which the responses of a neuron tuned to the target are divided by the responses of a population of other neurons (the normalization pool), including those that respond to the competitor. The competitor increases the responses of the normalization pool which suppresses responses to the target, similar to scaling the contrast of the target (i.e., changing the contrast gain of neurons that respond to the target).

There is also evidence that interocular suppression depends on attention, along with normalization. Ling and Blake [11] measured the detectability of a small monocular target in the presence of dichoptic competitors of different sizes. They found changes in behavioral performance implying that a large competitor induced a change in the contrast gain of neurons that responded to the target, whereas a small competitor induced a response-gain change (a change of the asymptotic response). The dependence on competitor size was analogous to that observed when manipulating spatial attention [12, 13]. Using an adaptation method, Ling and Blake further inferred that the response-gain modulation of the small competitor was absent when attention was withdrawn from the stimuli. Thus Ling and Blake [11] concluded that attention plays a major role in modulating competition in interocular suppression.

Whereas it has been proposed that attentional modulation plays a critical role in interocular suppression [11], it is unknown what type(s) of attention modulates the competition between the target and the competitor. Attention helps prioritize behaviorally relevant stimuli. In this study, we considered the role of spatial–, feature—and eye-based attention in interocular suppression. Attention can be driven by two different sources: goal driven—voluntary attention in response to task instructions that has a sustained effect (also known as endogenous attention), and stimulus-driven—involuntary attention in response to the abrupt onset of a salient stimulus (known as exogenous attention when elicited transiently by a brief stimulus). In this study, we used the terms goal driven and stimulus driven, instead of the terms endogenous and exogenous, because the stimulus was presented at fixation and its duration was not brief (1.5 s). When being deployed, attention can boost information at a given location (spatial attention [13–18]) or modulate the sensitivity of the sensory channels selective for relevant features; e.g., one of several orientations, motion directions, or colors (feature-based attention [19–25]). In addition, some studies have reported that, unbeknownst to the observer, attention can be eye specific [26,27].

We investigated the neural processing underlying interocular suppression using a combination of computational modeling and psychophysics. Our empirical results supported a model in which both attentional modulation and divisive normalization contributed to interocular suppression, and in which the effects of attention and normalization were clearly distinguishable from one another. Attention, in the best-fit model, was stimulus-driven, selective for both feature (orientation) and spatial location, but was not eye specific. Divisive normalization, on the other hand, can account for the change of contrast-gain modulation when the eye-of-origin of the stimuli was manipulated.

## Materials and Methods

### Ethical statement

The experiment was conducted with the written consent of each observer and the experimental protocols were approved by the University Committee on Activities involving Human Subjects at New York University.

### Psychophysics

Four observers (1 female and 3 males) participated in the main psychophysics experiment. All observers had normal or corrected-to-normal vision.

**Fig 1:**
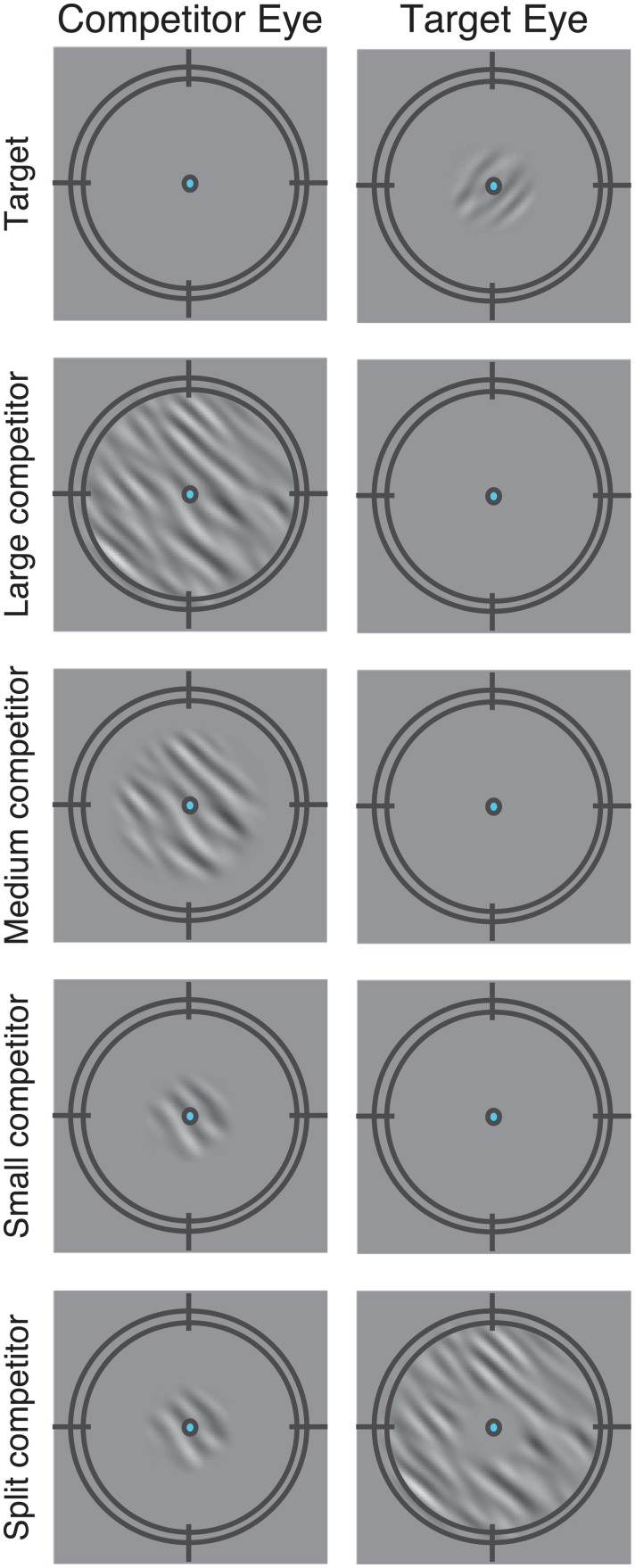
Stimuli. Band-pass filtered noise patches (1–6 cpd, 10° orientation bandwidth). Top row, a target presented to one eye. Middle rows, orthogonal competitors presented to the other eye. Bottom row, split competitor, parts of which were presented to each eye. Fixation point and black circular fusion frame were presented to both eyes throughout the experiment to stabilize the fixation and vergence so that the images presented to the two eyes would be aligned. In the main experiment, the right eye was the target eye and the left eye was the competitor eye. Two observers participated in additional experimental sessions in which the left eye was the target eye and the right eye was the competitor eye (See Psychophysics in Methods and Materials).

Stimuli consisted of band-pass filtered noise patches (1–6 cpd, 10° orientation bandwidth centered at 45° tilted clockwise from vertical), with a target presented to one eye (Fig 1, top row) and an orthogonal competitor presented to the other eye (Fig 1, middle rows; small, medium, and large competitors) or split between eyes (Fig 1, bottom row; split competitor). We chose the parameters to match those used in the previous study by Ling and Blake [11]. Target contrast varied from trial-to-trial in a randomly shuffled order, and the competitor contrast was fixed on every trial (23% RMS contrast). The different competitor configurations were interleaved in a randomly shuffled order within each block of trials. Each observer performed at least 50 trials for each of 45 conditions (a combination of nine target contrast levels and five types of competitors). The target and the small competitor were 1.5° in diameter, and the medium and large competitors were 2.5° and 8°, respectively. The split competitor (Fig 1, bottom row) had the same overall size as the large competitor except that it was segmented into two regions: the center (same size as the small competitor) was presented to the competitor eye while the surround was presented to the target eye. The two subregions of the split competitor combined as a uniform large stimulus when fused. The apertures of all the stimuli were smoothed with a Gaussian. The fixation point and a black circular fusion frame (9° x 9°) were presented to both eyes throughout the experiment to stabilize the alignment of the images presented to the two eyes. Stimuli were presented on a calibrated CRT monitor (75 Hz) positioned 57 cm from the observers. Observers viewed the screen through prism glasses, and a black cardboard septum ensured that the stimuli presented on the left half of the monitor were visible only to observer’s left eye and vice versa.

**Fig 2:**
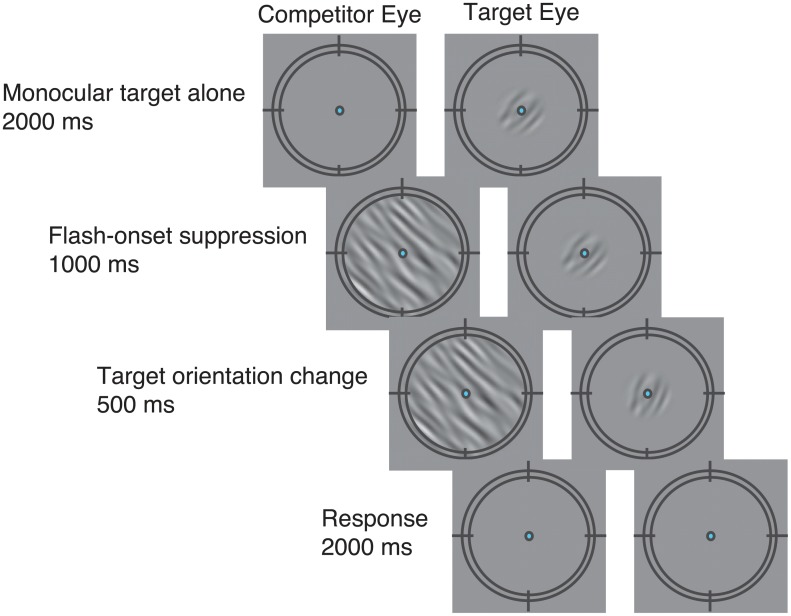
Example Trial Sequence. The target was first presented alone monocularly for 2 seconds followed by an abrupt onset of the competitor in the other eye. This onset-flash suppression procedure ensured that the competitor dominated the percept for a period of time following its onset. One second after the competitor onset, the target changed its orientation (4° clockwise or counterclockwise; for illustration purposes the orientation change is more pronounced here). Observers reported the direction of the orientation change by pressing one of two buttons.

Observers performed an orientation-discrimination task on the target (Fig 2). Each trial started with the target presented monocularly (to the target eye) for 2 s. The competitor was then added to the other eye, or to both eyes (for the split competitor). Typically, the competitor was dominant for a period of time following its onset [1]. One second after competitor onset, the target orientation changed either clockwise or counter clockwise (4°) for 500 ms and then both the target and the competitor disappeared from the screen. Observers reported the orientation change, clockwise or counterclockwise, by pressing one of two buttons. Psychometric functions—discriminability (*d*') versus target contrast—were measured for each observer and for each competitor configuration. This procedure, also known as onset flash suppression, allowed us to measure the visibility of the target when the target was suppressed and the competitor was dominant. Presenting the target and the competitor simultaneously (with simultaneous onset) and briefly, as in a masking experiment, would lead to a fused percept of the target and the competitor [28], unlike the strong competition of the two images observed in our experiment.

These parameters and procedures followed those in Ling and Blake’s experiments [11] except for two differences. First, we used a shorter interval (1 s) between the onset of the competitor and the orientation change of the target than the 2 s interval they used. In a pilot study, we found that the target reappeared frequently during the longer 2 s interval (unlike Ling and Blake, perhaps due to individual differences in temporal dynamics of interocular competition), which made it difficult to measure the suppression induced by the competitor. Second, we added the split competitor configuration which was critical for distinguishing between two alternative models.

In the main experiment, we presented the target to the observer’s right eye (the target eye) and the competitor, except the surround of the split competitor, to the observer’s left eye (the competitor eye), across all conditions. Two observers participated in additional experimental sessions in which three conditions (no competitor, large competitor and split competitor) were tested and the target eye and competitor eye were swapped: the target was presented to the left eye and the competitors (except the surround of the split competitor) were presented to the right eye. All the experimental procedures and parameters were the same as those used in the main experiment.

### Descriptive model

Psychometric functions were fit with Naka-Rushton functions to evaluate how different competitors influenced the visibility of the target:

$$d\prime (c)=d\prime_{m}\frac{c^{n}}{c^{n}+c_{50}^{n}}$$ (1)

where *d*'(*c*) was the behavioral performance as a function of target contrast, *d*'_*m*_ was the asymptotic performance at the highest contrast, and *c*_50_ was the semi-saturation constant determining the contrast level at which *d*' reached half the asymptotic performance. A change in *c*_50_ represented a contrast-gain change, and a change in *d*'_*m*_ indicated a response-gain change. The exponent *n* determined the slope of the function, and was constrained to have the same value for all competitor configurations. Allowing the slopes of the psychometric functions to vary across conditions yielded similar results that supported the same conclusions: the slopes were statistically indistinguishable across conditions, and the improvement of the goodness of fit was very subtle (*R* ^2^ improved by only 1.3%, on average across observers, and had a cost of adding five free parameters).

The statistical significances of the changes in contrast gain (*c*_50_) and response gain (*d*'_*m*_) were evaluated by a bootstrapping procedure. For each observer, we randomly resampled individual psychophysical trials with replacement to generate a bootstrapped data-set. Psychometric functions in the bootstrapped data-set were refit by Naka-Rushton functions. We then computed the differences between the group-averaged (across four observers) *c*_50_ and *d*'_*m*_ values obtained in the different competitor conditions. This procedure was repeated 2000 times to test whether the differences in *c*_50_ and *d*'_*m*_ values deviated significantly from zero. The statistical test and the confidence intervals of the estimated parameters for individual observers were obtained by the same procedure.

### Full model

**Fig 3:**
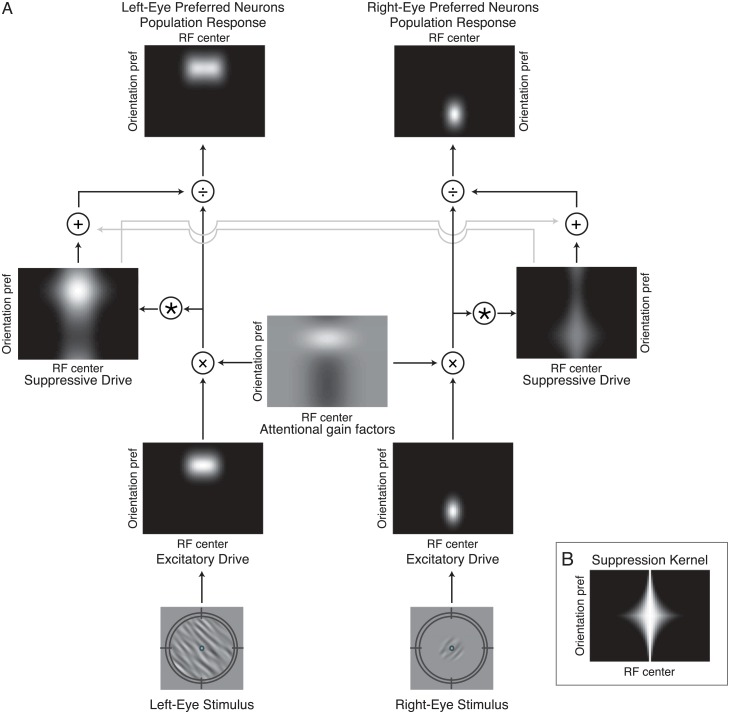
Model. **A**. The model simulates the firing rates of two populations of monocular neurons, responsive to stimuli presented to each of the two eyes, with a range of receptive field locations and orientation preferences. Depicted is an example of the feature-specific (FS) model, in which the two monocular neural populations share the same attentional gain factors, and stimulus-driven attention is selective for the orientation and size of the competitor. Bottom row, example stimuli. Target presented to right eye and large competitor presented to the left eye. Second row (from the bottom), excitatory drive. Simulated neurons are arranged in each panel according to their receptive field center (horizontal position) and preferred orientation (vertical position). Brightness at each location in the image corresponds to the excitatory drive to a single neuron. Third row, attentional gain factors. The attentional gains determine the strength of the attentional modulation as a function of receptive field center and orientation preference. Mid-gray indicates a value of 1, white indicates a value larger than 1 (attentional enhancement), and black indicates a value smaller than 1 (attentional suppression). Fourth row, suppressive drive computed from the product of the excitatory drive and the attention gain factors, and then pooled over space and orientation (see panel B), and across the two eyes (light gray arrows). Top row, output firing rates. The excitatory drive is multiplied by the attention gain factors and divided by the suppressive drive. **B**. The suppression kernel. The suppressive drive is pooled over space and orientation by convolving the attention-modulated excitatory drive with the suppression kernel.

We developed models to simulate the responses of a population of neurons to the stimuli used in the behavioral experiments (Matlab code is available on our website: http://www.cns.nyu.edu/heegerlab/). Each model had two neural populations: one preferred the stimuli presented in the left eye and the other preferred the stimuli in the right eye (Fig 3 and Table 1). For each of the neural populations, we simulated a 2-dimensional array of neurons, with orientation preferences uniformly sampling orientations in steps of 1° and receptive field centers ranging from -20° to 20°. The responses of the neurons (*R*) were determined by three components: the excitatory drive (*E*), the suppressive drive (*S*) and the attentional gain factors (*A*) [12]. Responses of the left-eye monocular neurons were computed by the following equation (the details of each component in the equation are listed in Table 1):

$$R_{L}(x,\theta )=[A_{x}(x,\theta )A_{v}(x,\theta )E_{L}{\left(x,\theta \right)}^{n}]/[S_{L}(x,\theta )+w_{I}S_{R}(x,\theta )+\sigma^{n}]$$ (2)

where *x* and *θ* represented the receptive field centers and preferred orientations of the neurons in the population, *n* was the exponent that controlled the slope of the neural contrast-response functions, *w*_*I*_ was the interocular normalization weight, and *σ* was a constant that determined the semi-saturation contrast. The value of *w*_*I*_ determined the contribution of inputs from the other eye to the normalization pool. The value of *σ* determined the contrast at which the neural responses saturated. The current model and parameterization only generated neural responses in positive numbers. We did not implement rectification or thresholding in Eq 2 because we did not simulate the effect of spiking threshold or neural responses below the baseline (zero in our case).

**Table 1:** Equations and parameter values for each of the components of the two models.

Model component	Equation	Description
Suppressive drive	$S_{L}=K_{S}*(A_{x}A_{v}E_{L}^{n})$	* denotes convolution. *K*_*s*_ is the suppression kernel. The attention gain factors, *A*_*x*_ and *A*_*v*_, and the excitatory drive $E_{L}^{n}$ were point-by-point multiplied.
Suppression kernel	$K_{S}(x,\theta_{i})=e^{\frac{-x^{2}}{2\sigma {\left(\theta_{i}\right)}^{2}}}$ $\sigma (\theta )=\sigma_{sx}e^{\frac{-\theta }{\tau }}$	*σ*_sx_ = 6°: width of the spatial suppressive field for iso-orientation stimuli. *τ* = 20°: orientation selectivity of surround suppression. Suppression was broadly tuned (all orientations) for stimuli within a neuron’s receptive field while surround suppression was narrowly tuned to the neuron’s preferred orientation. See Fig 1B.
Stimulus-driven attentional gain factors	$A_{x}=w_{x}(a_{\theta }a_{x}^{T})+1$	*w*_*x*_: free parameter that determined the strength of stimulus-driven attentional modulation. a_*θ*_ and a_*x*_: vectors representing the extent of feature-based and spatial attention. The attentional gain factors had a baseline of 1. Values larger/smaller than 1 indicated increases/decreases in gain.
Goal-driven attentional gain factors	$A_{v}=w_{v}(a_{\theta }a_{x}^{T})+1$	*w*_*v*_: free parameter that determined the strength of goal-driven attentional modulation. Both *A*_*x*_ and *A*_*v*_ involved computation of a_*θ*_ and a_*x*_ but the attended orientation and attended position were different for stimulus-driven and goal-driven attention (see Feature-based attention and Spatial attention below).
Feature-based attention	$a_{\theta }=e^{k\{\text{cos}\{2(\theta -\theta_{a})\}-1\}}-0.5$	A circular Gaussian (von Mises) profile scaled to range from -0.5 and 0.5. *θ*_*a*_: attended orientation. *k*: extent of feature-based attention. We used *k = 3*, which resulted in a 40° bandwidth (FWHM), similar to that used in previous simulations [37]. For stimulus-driven attention, *θ*_*a*_ was the orientation of the competitor in the FS model while *a*_*θ*_ was uniform (= 1) in the ES model. For goal-driven attention, *θ*_*a*_ was the orientation of the target in both FS and ES models.
Spatial attention	$a_{x}=\frac{1}{\sigma_{ax}^{p}\sqrt{2\pi }}e^{\frac{-{\left(x-x_{a}\right)}^{2}}{2\sigma_{ax}^{2}}}$	*p*: tradeoff between the spatial extent and the magnitude of attentional modulation. If *p* = 1, spatial attention had a fixed volume, decreasing in magnitude when its spatial extent increased. If *p* = 0, the spatial extent was independent of the magnitude of the attentional gain at the attended position *x*_*a*_. *σ*_*ax*_: spatial extent of spatial attention. For goal-driven attention, these parameters were fixed: *σ*_*ax*_ = 60° and *p* = 0. For stimulus-driven attention, *p* was a free parameter and *σ*_*ax*_ was equal to the size of the competitor.

The excitatory drive was determined by each neuron’s receptive field center and preferred orientation. The spatial excitatory field of each simulated neuron was a Gaussian with 1.5° standard deviation and the orientation tuning was a Gaussian with 48° FWHM (full width at half maximum), approximating the values reported for macaque primary visual cortex [29]. The suppressive drive was computed by pooling the excitatory drives of neurons with a range of receptive field centers and orientation preferences (See suppressive drive and suppression kernel in Table 1 and Fig 1). Suppression was broadly tuned (all orientations) for stimuli within a neuron’s receptive field whereas surround suppression was narrowly tuned to the neuron’s preferred orientation, mimicking electrophysiological findings [30,31]. To compute the neural responses, the excitatory drive of each neuron was multiplied by its attentional gain factor [12] and then divided by its suppressive drive [5,6,12,32]. Each neuron in the population had its own attentional gain factor that depended on the attentional state (i.e., the attended spatial position and the attended orientation) in particular experimental conditions. Two sources of attentional modulation were considered: *A*_*x*_ was the stimulus-driven attentional modulation induced by the competitor and *A*_*v*_ was the goal-driven attentional modulation induced by the demands of the orientation-discrimination task.

According to the model, both attention and normalization contributed to interocular suppression. First, the presence of the competitor induced stimulus-driven attentional modulation increasing the attentional gains of neurons tuned to the competitor and reducing the gains of neurons tuned to the target. Following Ling and Blake [11], this attentional modulation was responsible for the shift from a response-gain change to a contrast-gain change with increasing competitor size. Second, the competitor contributed directly to the normalization pool, weighted by the interocular normalization weight (*w*_*I*_ in Eq 2). This interocular normalization caused contrast-gain changes for all competitors, regardless of their size.

#### Stimulus-driven attentional modulation

We considered two possible ways to model the stimulus-driven attention induced by the competitor. Interocular divisive normalization was implemented in the same way in both models.

**Fig 4:**
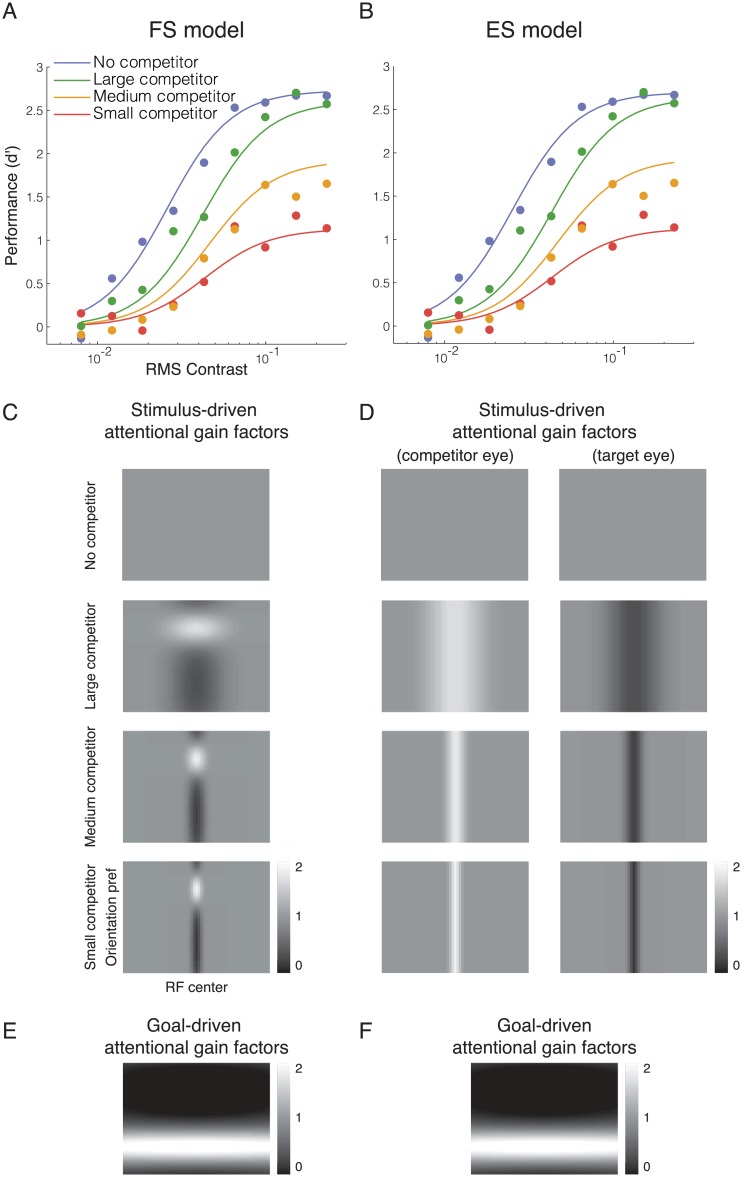
Model fits for data from Ling and Blake (2012). **A and B**. FS and ES model fits. Filled dots, psychophysical performance averaged across observers. Curves, best fits by each of the two models (parameter values reported in Table 2). **C and D**. The competitor-driven attentional gain factors estimated by the FS and ES models. **E and F**. The task-driven attentional gain factors estimated by the two models.

In the feature-specific model (FS model), the attentional gains were larger for neurons tuned to the orientation of the competitor and smaller for neurons tuned to other orientations (Fig 4C). The attentional gains had a spatial extent (*σ*_*αx*_ in Table 1) matching that of the competitor. The same attentional gain factors were applied to the right-eye and the left-eye neural populations.

In the eye-specific model (ES model), the attentional gains were larger for monocular neurons corresponding to the eye to which the competitor was presented, and smaller for neurons driven by the target eye (Fig 4D). The spatial extent of the attentional modulation was determined by the size of the competitor (as in the FS model), but there was no feature-based modulation in this model. For a given spatial location, the gain was increased by a multiplicative factor in one eye and reduced by the same multiplicative factor in the other eye.

Although the ES model may seem less plausible *a priori*, there is evidence for eye-specific attentional modulation. When non-perceptual artifacts are controlled, observers have no access to information about the eye-of-origin of a stimulus [33]. A few studies, however, have reported that eye-of-origin might impact attention unbeknownst to the observers. For example, visual search has been reported to be more efficient when the target and distractors were presented to different eyes [27]. It has also been reported that a monocular target under continuous flash suppression was more likely to become visible when observers simultaneously tracked a monocular stimulus presented to the target eye compared to when they tracked a stimulus presented to the other eye [26].

For both FS and ES model, a free parameter *p* controlled the trade-off between the spatial extent and the magnitude of the stimulus-driven attentional gain factors (see Spatial attention in Table 1). We also tested a compound model by fitting it to our data. In this model, the stimulus-driven attention had both feature-specific and eye-specific components (Supporting Information, S1 Text).

#### Goal-driven attentional modulation

In addition to the stimulus-driven attentional modulation induced by the competitor, both the FS and ES models included goal-driven attentional modulation induced by the task demands. Specifically, the attentional gains were larger for neurons tuned to the orientation of the target and the gains were smaller for neurons tuned to the orientation of the competitor (Fig 4E and 4F). This goal-driven feature-based attention was included in the models because it has been reported that observers deploy attention to orientation channels tuned to (or neighboring) the target orientation when performing a fine-grain orientation-discrimination task and when detecting a target superimposed with a high-contrast mask [34–37]. The spatial extent of the goal-driven attentional gain factors was assumed to be very wide in both models because both neuroimaging [38,39] and psychophysical [40,41] findings suggest that task-driven, feature-based attention spreads across space, influencing the neural responses or behavioral performance corresponding to uncued locations.

### Full model statistics

To fit the simulated neural responses to the psychophysics data, performance accuracy, *d*', was assumed to be proportional to the response of the neuron that was most responsive to the target (eye-of origin, orientation preference, and RF center). That is, we assumed additive, independent, and identically distributed (IID) noise [42]. We used a free parameter *σ*_*n*_, representing the magnitude of the noise, to relate behavioral performance (*d*') to the underlying neural responses. A change in response gain of the underlying neuronal responses thereby yielded a proportional scaling of the psychometric function and a change in contrast gain of the underlying neuronal responses yielded a proportional horizontal shift (on the log contrast axis) of the psychometric function. This interpretation of our behavioral results depended on the assumption of additive IID noise. However, given that the neurons homogeneously represent the sensory parameters and the tuning curve is invariant to contrast, an alternative model with Poisson noise and a maximum-likelihood decision rule would yield the same results; the performance (*d*') of an orientation decoder is proportional to the mean firing rate of the neuron within the neural population that is tuned for the mean stimulus orientation [43,44].

For both the FS and ES models, there were seven free parameters (See Table 2), and the MATLAB fmincon function was used to search for the best (least-squares) fit. All seven free parameters were fitted across all the conditions. The values of *w*_*x*_, *w*_*v*_ and *p* were constrained so that the attentional gain factors (*A*_*x*_ and *A*_*v*_) were non-negative. The only parameter that changed with different conditions was *σ*_*αx*_, which controlled the spatial extent of spatial attention (see Table 1). Both FS and ES models assumed that the spatial spread of stimulus-driven attention was determined by the competitor size so we set *σ*_*αx*_ to be the same as the width of the competitor (i.e., *σ*_*αx*_ was not a free parameter in the model fit). Confidence intervals for each of the free parameters were estimated by a bootstrapping procedure. Specifically, we randomly resampled individual psychophysical trials with replacement to generate a resampled data-set, which was refit with each of the models, and this procedure of resampling and refitting was repeated 2000 times to generate bootstrap distributions of the best-fit parameter values.

**Table 2:** Best-fit parameter values for data from Ling and Blake (2012).

Parameter	FS model	ES model	Description
	best-fit value [68% confidence interval]	best-fit value [68% confidence interval]	
*n*	2.16 [1.96 2.31]	2.01 [1.94 2.30]	Exponent of the neural contrast response function
*σ*	0.0020 [0.0019 0.004]	0.0019 [0.0018 0.004]	Constant term of the suppressive drive
*w*_*I*_	0.80 [0.18 0.94]	0.80 [0.06 0.94]	Interocular normalization weight
*w*_*x*_	4.70 [4.69 4.95] (1.00, 0.13, 0.20, 0.35)	2.41 [2.34 2.49] (1.00, 0.15, 0.28, 0.50)	Magnitude of stimulus-driven attentional modulation
*w*_*v*_	5.03 [4.95 5.02] (2.00)	4.99 [4.90 5.00] (2.00)	Magnitude of goal-driven attentional modulation
*p*	0.17 [0.16 0.35]	0.31 [0.18 0.41]	Trade-off between the magnitude and the spatial extent of the attentional gains
*σ*_*n*_	3.11 [2.62 3.08]	2.95 [2.61 3.07]	Magnitude of the noise
*R* ^2^	96.6%	95.9%	

For each parameter, we report the best-fit value and the 68% confidence interval obtained by a bootstrapping procedure. The value of *σ* is reported in units of excitatory drive (see Eq 2). In the row of *w*_*x*_, we also report the stimulus-driven attentional gain factor of the neuron tuned to the target in the no-, small-, medium- and large-competitor conditions (corresponding to the four values in the parenthesis respectively). In the row of *w*_*v*_, the goal-driven attentional gain factor of the neuron tuned to the target is reported too. Its value is the same across conditions because the spatial spread of goal-driven attention was constant across competitors (see details in Table 1).

We used cross-validation for model comparison. The raw data (a list of trials from each individual observer) were permuted and partitioned into a training set and test set, and the group-averaged psychometric functions were then computed for both the training set and the test set. Each model was fit to the training set to determine the best-fit parameters. These parameter values were then compared with the test set, i.e., by computing the coefficient of determination (*R* ^2^) which represented the goodness-of-fit of each model to the test set. The coefficient of determination was computed for the FS model ($R_{FS}^{2}$) and for the ES model ($R_{ES}^{2}$), and the difference between the two values ($R_{FS}^{2}-R_{ES}^{2}$) was taken as an index for model comparison. This procedure was repeated 2000 times to obtain a distribution of $R_{FS}^{2}-R_{ES}^{2}$ values. The FS model was considered to outperform ES model if this distribution was significantly (95% of the distribution) larger than zero, and the ES model was considered as the better model if the distribution was significantly smaller than zero. The model fit and the model comparison were performed on the group averaged data consisting of 11079 trials pooled across observers and conditions. In a complementary analysis, we also used maximum-likelihood estimation and Bayesian information criterion to compare the models (Supporting Information, S2 Text).

To assess whether there was parameter redundancy, we numerically computed the Hessian matrix (i.e., the second derivatives with respect to the model parameters) of the best-fit model, and used singular value decomposition to compute the rank of the matrix. A full-rank Hessian matrix indicated that the parameters in the model were not redundant [45].

## Results

### Both models fit Ling and Blake (2012)

To investigate the role of attention and normalization in interocular suppression, we developed computational models to simulate the responses of a population of neurons, and fit the models to published psychophysical measurements [11]. Ling and Blake [11] reported changes in behavioral performance implying that a large competitor induced a change in the contrast gain of neurons that responded to the target, whereas a small competitor induced a response-gain change. Two models were considered as candidates. In the feature-specific (FS) model, the onset of the competitor induced stimulus-driven attentional modulation that increased the gain of neurons that preferred the competitor orientation, and reduced the gain of neurons that responded preferentially to the target orientation. In the eye-specific (ES) model, stimulus-driven attentional modulation increased the gain of neurons that responded preferentially to inputs from the eye to which the competitor was presented, and reduced the gain of neurons that responded to the target eye. In both models, the stimulus-driven attentional modulation was complemented by goal-driven attentional modulation that increased the gain of neurons that preferred the target orientation, and reduced the gain of neurons that preferred the competitor orientation. This goal-driven attention component was task-specific, enabling the simulated observer to discriminate the target orientation more accurately. Divisive normalization also contributed to interocular suppression in both models.

Both models were able to explain the suppression induced by the competitor in Ling and Blake’s data (Fig 4). The models were fit to the data to determine best-fit values for the model parameters including the magnitudes of the stimulus-driven and goal-driven attentional modulations, and the interocular normalization weight (Table 2). There was no evidence for a difference between the goodness of fit of the two models (p = 0.45; see Materials and Methods, Full model statistics).

According to both models, behavioral performance depended on competitor size because the onset of the competitor induced stimulus-driven attentional modulation with a spatial extent determined by competitor size (Fig 4C and 4D). The parameter *p* (Table 2) represented the tradeoff between the magnitude and spatial extent of the stimulus-driven attentional gain factors. A value of *p* close to 1 would have indicated that the sum of the attentional gain factors was constant when size varied, i.e., a complete trade-off between size and magnitude. The best-fit values were 0.17 and 0.31 for the FS and ES models, respectively, indicating a partial trade-off between size and magnitude. Due to this tradeoff, in addition to the spatial extent of attention, the values of the (stimulus-driven) attentional gain factors also changed with the size of the competitor (see the values reported in the parenthesis under *w*_*x*_ in Table 2). The model fit also showed a significant role of goal-driven attention (*w*_*v*_ in Table 2) which increased the gain of neurons tuned to the target orientation and reduced the gain of neurons tuned to the competitor orientation (Fig 4E and 4F).

The interocular normalization weight *w*_*I*_ was around 0.8 for both FS and ES models (Table 2), implying nearly equal divisive normalization from stimuli presented to either eye. However, the confidence interval for this parameter value covered a wide range from 0 to 1. There was no evidence of parameter redundancy in the model fits because the Hessian matrices were full rank (see Full model statistics in Materials and Methods). However, the interpretation of interocular normalization weights could be difficult because the eye-of-origin of the competitor was not manipulated in Ling and Blake’s experiment, and the magnitude of the interocular divisive normalization did not solely depend on this parameter but also on the goal-driven attentional modulation which could reduce the gain of the responses evoked by the competitor. The role of interocular divisive normalization and how it influenced the predicted psychometric functions became clearer when the eye-of-origin of the competitor was manipulated in our psychophysics experiment.

### Distinguishing between the two models with the split competitor

To distinguish the FS model and the ES model, we replicated Ling and Blake’s (2012) psychophysics experiment and added a critical new condition with a split competitor. The split competitor (Fig 1, bottom row) had the same overall size as the large competitor except that it was segmented into two regions: the center (same size as the small competitor) was presented to the competitor eye whereas the surround was presented to the target eye. The two subregions of the split competitor were perceived as a single large grating when fused. The FS model predicted that the split competitor would cause a contrast-gain change like the large competitor; according to this model, the split competitor induced the same attentional modulation as that driven by the large competitor because the attentional gain factors were blind to the eye-of-origin of the stimuli. The ES model predicted that the split competitor would cause a response-gain change like the small and medium competitors; according to this model, the split competitor induced less attention in the center region of the target eye (because the center of the competitor was presented to the other eye) and more attention in the surround region of the target eye (because the surround of the competitor was presented to the target eye). In addition, we predicted that the split competitor condition would constrain the best-fit value of the interocular normalization weight *w*_*I*_ because the large competitor and the split competitor had the same overall size but differed in presentation to the two eyes.

#### Descriptive model

**Fig 5:**
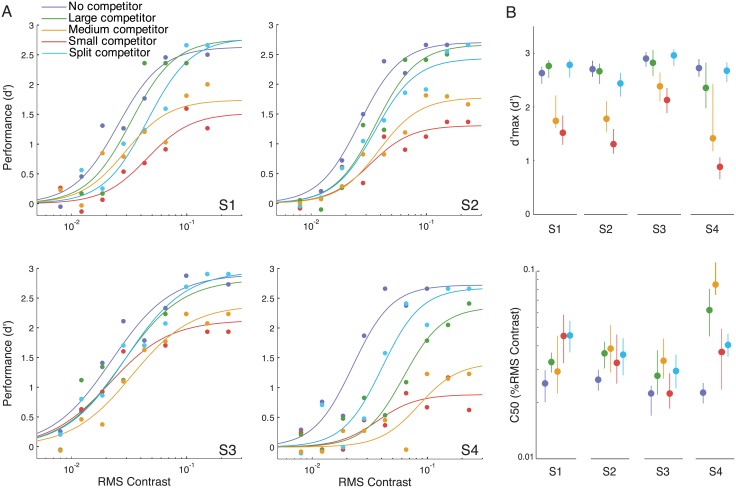
Descriptive model fits. **A**. Psychometric functions for each individual observer. Filled dots, psychophysical performance. Curves, best fit Naka-Rushton functions. **B**. Best-fit *c*_50_ and *d*'_*m*_ parameter values for each individual observer. Error bars, bootstrapped 95% confidence intervals.

The split competitor shifted the psychometric function commensurate with a contrast-gain change, like the large competitor (Fig 5). A descriptive fit to the data confirmed that the split competitor changed the contrast-gain parameter *c*_50_ (*p*<0.001, bootstrap test; see Materials and Methods, Descriptive model statistics) without reliably changing asymptotic performance *d*'_*m*_ (*p* = 0.214). These results were consistent across observers (change in *d*'_*m*_: *p*>0.1 for all observers; change in *c*_50_: *p*<0.05 for S1,S2 and S4; *p* = 0.07 for S3; bootstrap tests). For the other conditions, our results were consistent with those reported by Ling and Blake (2012): the small and medium competitors induced response-gain changes (both *p*<0.001), and the large competitor induced a contrast-gain change (*p*<0.001) without reliably changing asymptotic performance (*p* = 0.112).

The split competitor and large competitor masked the target by comparable amounts, on average. The best-fit values for the contrast-gain change parameter *c*_50_ were statistically indistinguishable between these two conditions (*p* = 0.271). There were, however, notable individual differences in the best-fit values for *c*_50_. For observer S4, the shift in *c*_50_ was larger for the large competitor than the split competitor (*p*<0.01, bootstrap test). Observer S1 showed the opposite: larger *c*_50_ shift for the split-competitor (*p*<0.05, bootstrap test). For the other two observers, there was no evidence for a difference.

#### Full model

**Fig 6:**
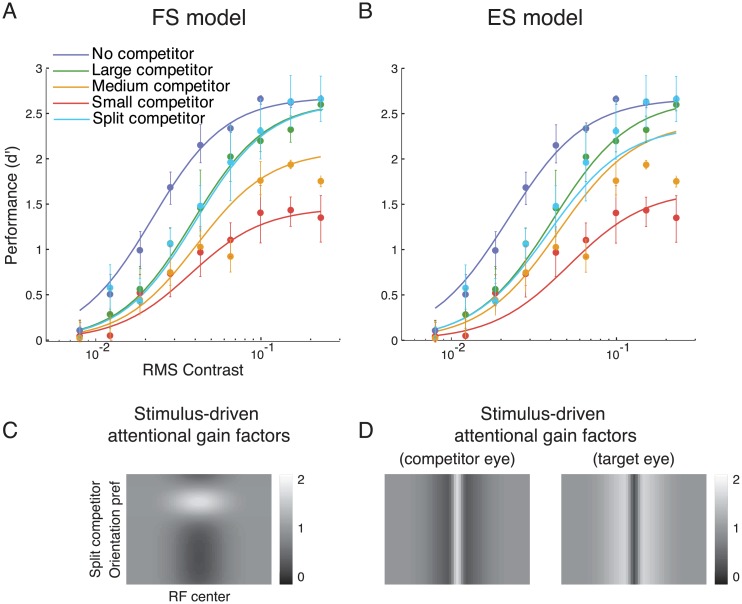
Full model fits. **A** and **B**. FS and ES model fits. Filled dots, psychophysical performance averaged across observers. Error bars represents ±1 SEM. Curves are the best-fit *d*'(*c*) by each of the two models (parameter values reported in Table 3). **C and D**. Stimulus-driven attentional gain factors for the split-competitor condition, estimated by each of the two models. The stimulus-driven attentional gain factors for the other conditions and the goal-driven attentional gain factors are similar to those reported in Fig 4.

The FS model outperformed the ES model (Fig 6). Both models captured the contrast-gain change for the large competitor and the response-gain change for the small competitor. However, the ES model underestimated performance for the split competitor and over-estimated performance for the medium competitor. Statistical comparison between the model fits was done with cross validation, randomly resampling from the data 2000 times (see Materials and Methods, Full model statistics). The FS model provided a consistently better fit than the ES model (P<0.0005; see Materials and Methods, Full model statistics). Indeed, for all 2000 re-sampled datasets, the *R* ^2^ values obtained with the FS model were larger than those obtained with the ES model. When we applied maximum likelihood estimation and used Bayesian information criterion as the index for model comparison, we again found that the FS model was superior to the ES model (Supporting Information, S2 Text).

We also considered a compound model in which stimulus-driven attention had both feature-specific and eye-specific components. We did not find notable improvement by including both components in the model. In the best-fit parameters, the magnitude of the eye-specific attentional modulation was reduced to a value close to zero (Supporting Information, S1 Text).

The best-fit parameter values for the new data set were generally compatible with those for the Ling and Blake (2012) data set (compare Tables 2 and 3). One exception was that the trade-off parameter *p* for the ES model had a larger value for our data (0.707, 68% CI: [0.704 0.856]) than for Ling and Blake’s data (0.312, 95% CI: [0.175 0.412]). When *p* was fixed at the best-fit values for Ling and Blake’s data (Table 2), the ES model provided an even worse fit to the split competitor condition, because it predicted an even larger response-gain change, similar to that observed for the small competitor. The best-fit values of *p* for the FS model were indistinguishable between the two data sets. As expected, when the split competitor was included, the data constrained the estimated *w*_*I*_ to have a narrower range (CI of *w*_*I*_ in FS model: [0.460 1.043], Table 3) compared to the previous model fit (CI of *w*_*I*_ in FS model: [0.175 0.940], Table 2).

**Table 3:** Best-fit parameter values for group-averaged data.

Parameter	FS model	ES model	Description
	best-fit value [68% confidence interval]	best-fit value [68% confidence interval]	
*n*	1.95 [1.84 2.08]	1.85 [1.72 1.95]	Exponent of the neural contrast response function
*σ*	0.0016 [0.0015 0.0033]	0.0016 [0.0015 0.0032]	Constant term of the suppressive drive
*w_I_*	0.67 [0.46 1.04]	1.08 [0.87 1.03]	Interocular normalization weight
*w_x_*	4.24 [4.10 4.34] (1.00, 0.20, 0.26, 0.36, 0.36)	2.46 [2.37 2.57] (1.00, 0.26, 0.49, 0.77, 0.77)	Magnitude of stimulus-driven attentional modulation
*w_v_*	5.03 [5.01 5.03] (2.01)	4.90 [4.82 4.93] (1.98)	Magnitude of goal-driven attentional modulation
*p*	0.13 [0.11 0.22]	0.71 [0.70 0.86]	Trade-off between the magnitude and the spatial extent of the attentional gains
*σ_n_*	2.92 [2.75 3.08]	2.82 [2.59 2.93]	Magnitude of the noise
*R* ^2^	97.1%	94.8%	

For each parameter, we report the best-fit value and the 68% confidence interval obtained by a bootstrapping procedure. The value of *σ* is reported in units of excitatory drive (see Eq 2). In the row of *w*_*x*_, we also report the stimulus-driven attentional gain factor of the neuron tuned to the target in the no-, small-, medium-, large- and split-competitor conditions (corresponding to the five values in the parenthesis, respectively). In the row of *w*_*v*_, the goal-driven attentional gain factor of the neuron tuned to the target is reported too. This value is the same across conditions because the spatial spread of goal-driven attention did not change with competitor (see details in Table 1).

The individual differences in the amount of suppression induced by the split competitor (in comparison to that induced by the large competitor) were accounted for by individual differences in the interocular normalization weight *w*_*I*_. The FS model was fit to the data from each observer individually to determine the best-fit parameter values (Table 4). For observers S2 and S3, who exhibited roughly the same suppression for the large and split competitors, the best-fit values for *w*_*I*_ were close to 1. The *w*_*I*_ value was much larger than 1 for observer S4, who exhibited a stronger contrast-gain change for the large competitor than the split competitor. The *w*_*I*_ value was much smaller than 1 for observer S1, who exhibited a weaker contrast-gain change for the large competitor than the split competitor.

**Table 4:** Best-fit parameter values for individual observers.

Parameter	S1	S2	S3	S4	Description
*n*	2.15	2.21	1.67	2.22	Exponent of the neural contrast response function
*σ*	0.0019	0.0018	0.0014	0.0016	Constant term of the suppressive drive
*w_I_*	0.01	1.08	1.15	3.81	Interocular normalization weight
*w_x_*	4.62	4.50	2.96	4.89	Magnitude of stimulus-driven attentional modulation
*w_v_*	4.65	5.03	5.02	5.03	Magnitude of goal-driven attentional modulation
*p*	0.26	0.20	0.24	0.12	Trade-off between the magnitude and the spatial extent of the attentional gains
*σ_n_*	3.10	3.26	2.49	3.18	Magnitude of the noise
*R* ^2^	91.7%	94.6%	91.3%	89.2%	

The value of *σ* is reported in units of excitatory drive (see Eq 2).

### Eye-of-origin modulates the magnitude of contrast gain

In the experiment above, we always presented the target in the observer’s right eye. To further investigate the role of eye-of-origin and its relation with the interocular normalization weight parameter *w*_*I*_, two observers (S1 and S4), who showed very different contrast gain changes in the large- and split-competitor conditions, participated in additional experimental sessions in which the target was presented to the left eye and the competitors were presented to the right eye (except the surround of the split competitor). The no-competitor, large-competitor and split-competitor conditions were tested. The comparison between the modulations induced by the large competitor and the split competitor could reveal the role of eye-of-origin because these two conditions had the same overall (perceived) competitor size, and the only difference between the two competitors was how the competitor was distributed between the two eyes.

**Fig 7:**
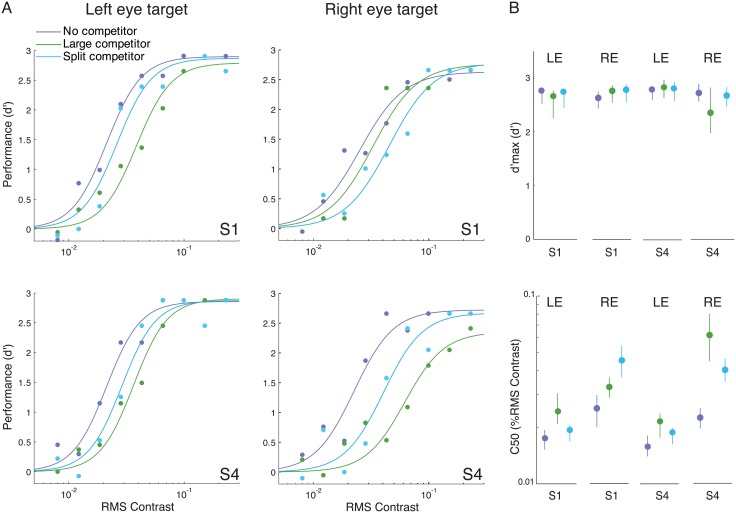
Descriptive model fits. **A**. Psychometric functions for two individual observers who participated in additional experimental sessions, in which the target was presented to the left eye and the competitor presented to the right eye. The corresponding data for right-eye targets are the same as the data reported in Fig 5. Filled dots, psychophysical performance. Curves, best fit Naka-Rushton functions. **B**. Best-fit *c*_50_ and *d*'_*m*_ parameter values for each individual observer. LE represents left-eye target, and RE represents right-eye target. Error bars, bootstrapped 95% confidence intervals.

When the target eye and competitor eye were swapped, both split competitor and large competitor still induced a contrast gain change (large competitor: *p*<0.001, split competitor: *p*<0.01, bootstrap test) without modulating the response gain (large competitor: *p* = 0.69, split competitor: *p* = 0.46, bootstrap test) (Fig 7, left column). S1, who originally showed stronger contrast gain modulation in the split-competitor condition, now had stronger modulation in the large-competitor condition (*p*<0.05, bootstrap test; Figs 5 and 7). For S4, the large competitor generated stronger suppression than the split competitor in the original experiment, but the suppressive effects of the two conditions became indistinguishable when the target eye and competitor eye were swapped (*p* = 0.10, bootstrap test).

When fitting the FS model to individual data, *w*_*I*_ again reflected these individual differences. For S1, *w*_*I*_ changed from 0.01 (Table 4) to 1.98 (Table 5). For S4, *w*_*I*_ reduced from 3.81 to 0.71. The increase of *w*_*I*_ indicated stronger suppression induced by the stimulus presented in the competitor eye than in the target eye. The change of *w*_*I*_ value in the model fit followed the results that after swapping the eye-of-origin, the large competitor (which was presented entirely in the competitor eye) became more suppressive for S1 and less suppressive for S4. The fitted value of *p* was larger in Table 5 than that in Table 4. However, the value of this parameter should be interpreted with care. In the model fitting process of the main experiment, we found that the value of *p* was mainly determined by the amount of response gain reduction observed in the small and medium competitors (for example, a larger trade off between the magnitude of attentional modulation and the spatial spread of attention would let the response gain of the medium competitor be closer to that of the large competitor, and vice versa). Here, we only fit the no competitor, large competitor and split competitor conditions. None of these conditions exhibited response gain modulation, so the data might not have constrained the value of *p*. To determine if this was the case, we assessed the Hessian matrix of the best-fit model, for each subject. While the Hessian matrices were full rank, the eigenvectors corresponding to the smallest eigenvalues had a large projection (0.64 for S1 and -0.85 for S4) on parameter *p*, implying that this parameter was not as well-constrained as the other parameters.

**Table 5:** Best-fit parameter values for individual observers.

Parameter	S1	S4	Description
*n*	2.69 (2.76)	2.71 (2.92)	Exponent of the neural contrast response function
*σ*	0.002 (0.002)	0.002 (0.002)	Constant term of the suppressive drive
*w_I_*	1.98 (0.83)	0.71 (0.12)	Interocular normalization weight
*w_x_*	3.31 (fixed: 4.62)	2.63 (fixed: 4.89)	Magnitude of stimulus-driven attentional modulation
*w_v_*	5.03 (5.04)	5.01 (5.04)	Magnitude of goal-driven attentional modulation
*p*	0.80 (fixed: 0.26)	0.67 (fixed: 0.12)	Trade-off between the magnitude and the spatial extent of the attentional gains
*σ_n_*	3.45 (3.52)	3.52 (3.63)	Magnitude of the noise
*R* ^2^	95.3% (94.7%)	97.0% (95.9%)	

FS model was fit to the individual data in which the target eye and competitor eye were swapped. The values in the parenthesis are the fitted values by a constrained model in which the *w*_*x*_ and *p* are fixed at the values reported in the main experiment.

We fixed the parameters related to stimulus-driven attention, *w*_*x*_ and *p*, at the values reported in the main experiment and fit the model again (parenthesis in Table 5). We found that the FS model could still account for the data, and the *R* ^2^ only decreased by 0.59% and 1.14% for S1 and S4 respectively. The change of *w*_*I*_ due to swapping the eye-of-origin was in the same direction as the original model fit (increase for S1 and decrease for S4 compared to the main experiment).

## Discussion

We proposed a computational model integrating attentional modulation and divisive normalization to explain the contrast-gain and response-gain modulation in interocular suppression. The form of interocular suppression (contrast- versus response-gain change) depended on the size of the competitor (not the eye to which it was presented), thereby supporting a model in which attention was selective for the orientation and spatial location of the competitor, not the eye-of-origin of the competitor. The eye-of-origin of the competitor influenced only the magnitude of the contrast-gain change, which the model accounted for with interocular divisive normalization.

### Stimulus-driven feature-based attention

In the normalization model, the responses of a visual neuron are modulated by both the neuron’s preferred stimulus and the visual context formed by the presence of other stimuli [6]. Several models of visual attention have suggested that the effect of attention can be modeled within the framework of normalization [46–48]. In the present study, we followed the normalization model of attention proposed by Reynolds and Heeger [12]. This model correctly predicts that endogenous and exogenous spatial attention cause contrast-gain and response-gain changes, evident in the psychometric functions, when the spatial extent of attention is manipulated [13], and response-gain changes when the featural extent of attention is manipulated [37].

In the present experiment, the competitor elicited an abrupt visual onset and thus stimulus-driven attention [25,49–52]. According to the model, stimulus-driven attention influences behavioral performance even when it opposes task demands (in this case, monitoring the orientation of the target), and implies that the bottom-up inputs have a considerable impact on the deployment of feature-based attention. This is different from most previous studies, in which feature-based attention was presumed, or manipulated, as a top-down process controlled voluntarily by observers [24,34,39,53].

The stimulus-driven, feature-based attention seems to have properties different from goal-driven feature-based attention. For example, the spatial extent of the stimulus-driven feature-based attention was constrained by the size of the competitor stimulus. In contrast, previous studies have found that goal-driven feature-based attention spreads across space [38–40, also see 54,55]. The partial trade-off that we observed between the magnitude of stimulus-driven attention and its spatial extent is consistent with the idea of limited attentional resources [16,24,56].

One study also found a significant influence of bottom-up inputs on feature-based attention by showing a reduction of reaction time when the pre-cue and the target to be discriminated were in the same color compared to when they were different [25]. The authors reported that this featural effect can spread across space, but only when the cue was presented in one of the potential target locations and not when it was presented in a target-irrelevant location. To explain the results of the present experiment, we infer that stimulus-driven feature-based attention followed the size of the competitor even when it extended beyond the task-relevant target location. The difference between our and Lin et al.’s [25] results might be due to the discrepancy of the experimental designs between the two studies. The response time measure used by Lin et al. could be influenced by the target’s visibility, the speed of information processing and/or criteria changes [57,58]. On the other hand, in the present study, we focused on the visibility of the target by using *d*' as the index.

Feature-based attention with benefits for attended features and costs for unattended features reported in our model is consistent with previous behavioral [40] and neurophysiological [59] studies. Even though the increases and reductions in attention gain were linked together in our model, we are agnostic as to whether they share a unified mechanism. Multi-unit recordings in FEF and V4 in monkeys [60] and human EEG experiments [61] have shown that such increases and reductions in attention gain occur with different time courses.

Our psychophysical results and model comparison showed that the attentional modulation in interocular suppression was better described by a feature-specific rather than an eye-specific modulation. Solely based on our results, we can not conclude that attention is unable to modulate eye-specific information. Previous studies proposing eye-based attention found that manipulating the eye-of-origin of the stimuli [27], or the eye-of-origin of the image to be attentively tracked [26], can influence observers’ performance in a visual task. However, in our experiment and computational model, we also identified a source of suppression, in addition to attention, whose strength depended on the eye-of-origin of the stimuli (see Interocular divisive normalization below). Many studies [2,7,9,62] have also shown that the magnitude of suppressive interactions between multiple stimuli can be changed by simply manipulating eye-of-origin (presented simultaneously either in the same eye or different eyes). Future studies should establish whether the eye-based attention effects reported earlier are distinguishable from this eye-dependent suppression.

### Goal-driven attention

The goal-driven component of attention in our model is similar to that in previous models of feature-based attention in orientation discrimination tasks [23,35–37]. Indeed, there is evidence that feature-based attention also modulates interocular suppression [63]. Because goal-driven attention was the same for all of the stimulus conditions, it could not account for the dependence of competitor size on interocular suppression. In preliminary fits to Ling and Blake’s data set, we found that without goal-driven attention the interocular normalization weight *w*_*I*_ was forced to take an extremely small value. However, a *w*_*I*_ value close to zero predicted that the split competitor should induce a much stronger contrast-gain change than any of the other competitors, contrary to what we observed (Figs 5, 6 and 7). Including goal-driven, feature-based attention is, consequently, in accordance with the feature-based attention literature and allows a unified model that can fit the results of both experiments.

Other studies have modeled the demands of the task by including a weighting function at the decoding or decision stage of processing [64–66]. We acknowledge that the goal-driven gain factors in our current model might be replaced by multiple stages of information processing, and this could be the reason why the goal-driven gain factors estimated by the model were so strong that they greatly reduced the response of the task-irrelevant orientations (Fig 4E).

### Interocular divisive normalization

We found, for some individuals, that moving a portion of the competitor to the other eye (changing the stimulus from large competitor to split competitor) can influence the magnitude of contrast-gain change without influencing the response gain. This pattern held when the target eye and competitor eye were swapped so that the target was presented to the left eye and the competitors were presented to the right eye (Fig 7). These results provided evidence that a source of the suppression from the competitor is eye-specific and it causes a change in contrast gain, consistent with previous neuroimaging [9] and psychophysics [8] studies on cross-orientation dichoptic masking.

In the current model, the interocular normalization weight *w*_*I*_ reflected individual differences in the magnitude of the contrast-gain change induced by the large and the split competitors: increasing the value of *w*_*I*_ caused the large competitor to become more suppressive compared to the split competitor. The individual variation in the magnitude of interocular divisive normalization was consistent with previous studies reporting individual variations in the strength and the temporal dependency of dichoptic masking [7,62]. There seem to be multiple factors influencing the magnitude of *w*_*I*_: Individual differences in eye dominance are well documented for normal observers [67, 68]. In the present experiment, if an observer had a significant imbalance in eye dominance, moving a portion of the competitor from the weaker eye to the stronger eye should have resulted in greater divisive suppression of the target. In addition, after swapping the target eye and the competitor eye, the competitor (either the large or the split competitor) that generated stronger suppression should have also switched (to the split or to the large competitor, respectively). We observed this pattern for subject S1, but not for S4 (Fig 7). Thus, the magnitude of interocular normalization can not be solely explained by eye dominance. The model fits (S1 and S4 in Tables 4 and 5) indicated that the interocular suppression weight from left- to right- eye was not equal to the weight from right- to left- eye, and thus *w*_*I*_ not only varied across observers but also varied according to which eye was the target eye (and which eye was the competitor eye). The parameter *w*_*I*_ can actually be considered to be two separate parameters: *w*_*LR*_ in Table 4 and *w*_*RL*_ in Table 5, such that *w*_*LR*_ represents the strength of interocular divisive normalization contributed by the left-eye competitor to the right-eye neural population, and vice versa for *w*_*RL*_.

### Dissociable roles of attention and normalization

There has been some controversy as to whether interocular suppression occurs in early visual cortex when attention is controlled. Whereas one study reported an absence of interocular suppression in V1 [69], two studies reported significant interocular suppression even when attention was diverted away from the stimuli [9,10]. According to the current model, both divisive normalization and attentional modulation contribute to interocular suppression. We demonstrated that these two components contributing to interocular suppression can be distinguished. Attentional modulation depended on the feature and size of the competitor resulting in a response-gain change for large competitors and a contrast-gain change for small competitors. Interocular divisive normalization depended on the eye-of-origin of the competitor resulting in contrast-gain changes of different magnitude for the large and split competitors.

### Comparison with previous models and model limitations

Previous psychophysics studies [7,8] investigated the suppression induced by dichoptic masks with orientation orthogonal to the target and the same size as the target. It was found that the mask elevated detection threshold of the target and the data were fitted by a model with interocular divisive normalization [7]. When a large range of target contrasts were tested, a contrast gain modulation was reported in a contrast detection task [8]. Such a pure contrast gain modulation is different from the suppression effect reported here. The discrepancy might be due to the fact that the interocular suppression was measured in distinct perceptual states. Our study used onset-flash suppression to ensure that the target was suppressed by the dominant competitors. This is different from the procedures used by Baker et al. [7,8], in which the target and the mask were presented simultaneously and briefly (from 25 ms to 400 ms). These parameters are known to generate a perceptual state of fusion (of the target and the mask) before the initiation of strong interocular competition [28].

Single-cell electrophysiological recordings have found mixed results regarding the suppression induced by dichoptic masks. Some reported predominately response gain modulation [70], and some reported a mixture of contrast gain and response gain modulation varying across neurons [71]. These experiments were conducted on anesthetized and paralyzed cats and one should be careful when linking these results with neuroimaging and psychophysics on humans. A series of studies have reported strong dependency between the animals’ states and the response of neurons in primary visual cortex including visually evoked response [72], magnitude and correlation of noise [73] and suppressive connections [74]. Electrophysiological and neuroimaging studies on monkeys and humans have shown divergent results when probing the neural correlate of interocular suppression in V1, either with the same or different types of neural signal and measurement [reviewed in 4]. Attentional state is a critical factor when comparing the results across experiments because interocular suppression is dependent on attentional state [11,75,76].

Two human neuroimaging studies [9,10] reported dichoptic masking effects in V1 and showed that the suppression could be accounted for by divisive normalization. These experiments used a central attention task to control observers’ attention at fixation. This is generally consistent with the prediction of the current model that if attention is withdrawn from the stimuli, the competitor will only generate a contrast gain change which can be accounted by interocular divisive normalization. Single-cell recording [77] and psychophysical [7] studies have shown that the origins of the suppression contributed by monocular and dichoptic masks can be distinguished by manipulating the spatiotemporal properties of the mask or by testing the adaptability of the mask. In the current model, the stimuli presented either in the same or different eyes can suppress the neurons through normalization. We did not aim to address the particular neural pathways that support the normalization in the same eye or across eyes; normalization might occur at different stages of neural processing and it might be implemented by different neural mechanisms [6].

In dichoptic masking studies, interocular suppression can be reduced by adding matched images, with either non-zero [78] or zero [79] disparity, in the two eyes. In contrast, in our experiment, presenting the split competitor to both eyes did not increase the correspondence between the images in the two eyes. Likewise, adding binocular fusion markers to manipulate the correspondence between the images in the two eyes did not change the size-dependent interocular suppression [11].

In binocular rivalry, it has been reported that a large stimulus presented to one eye has shorter dominance durations than that of a small stimulus presented simultaneously to the other eye [80], similar to the size effect reported here. We are agnostic about whether the surface-boundary account proposed by Ooi and He [80] to explain this binocular rivalry result could be used to model the contrast-gain and response-gain modulation in our experiments and the absence of response-gain modulation by the small competitor under withdrawn attention reported by Ling and Blake [11].

In addition to the onset-flash suppression used in this study, various related methods have been used to probe the interocular interactions regulating competing information from the two eyes, e.g., binocular rivalry [81], generalized flash suppression [2], and continuous flash suppression [3]. Our model does not attempt to account for these various perceptual phenomena. For example, in continuous flash suppression, presenting a static target with a dynamic competitor gives rise to a depth of suppression much stronger than most of the other methods of interocular suppression. How such manipulations increase the dominance duration is beyond the scope of present study. Even so, it is likely that these distinct perceptual phenomena share common neural processes [8,82]. Further research is required to investigate whether the model proposed here, including divisive normalization, as well as spatial- and feature-selective attention, can be extended to explain this wide range of perceptual phenomena.

## Supporting Information

S1 Text Compound model. (PDF)

S2 Text Model comparison by maximum likelihood estimation and Bayesian information criterion. (PDF)
