# Supplementary material for: Deconstructing Interocular Suppression: Attention and Divisive Normalization
Source: PLoS Comput Biol. 2015 Oct 30;11(10):e1004510. doi: 10.1371/journal.pcbi.1004510 (PMC4627721; doi:10.1371/journal.pcbi.1004510)
Supplement: S1 Text — (PDF) [file pcbi.1004510.s001.pdf]

Deconstructing Interocular Suppression:
Attention and Divisive Normalization

Hsin-Hung Li[1], Marisa Carrasco[1,2], David J. Heeger[1,2*]

PLOS Computational Biology
(2015)

[1] Department of Psychology, New York University, New York, New York 10003
[2] Center for Neural Science, New York University, New York, New York 10003
* Corresponding author: David J. Heeger, Department of Psychology, New York University, New York, New York 10003, E-mail: david.heeger@nyu.edu

S1 Text. Compound model

In the compound model, the stimulus-driven attentional modulation had both a feature-specific component, A_{xF}, and an eye-specific component, A_{xE}. The two components were computed in the same way as the stimulus-driven attentional gain factor described in the FS model and ES model in the main text. Responses of the left-eye monocular neurons in the compound model were computed by the following equation:

$$R_L(x,\theta) = [A_{xF}(x,\theta)A_{xE}(x,\theta)A_v(x,\theta)E_L(x,\theta)^n] / [S_L(x,\theta) + w_I S_R(x,\theta) + \sigma^n]$$

which is the same as Equation 2 in the main text, except that the stimulus-driven attentional modulation was composed of two components. The parameterization of the compound model was the same as for the FS and ES models, but the compound model had one additional free parameter (8 in total, listed in Table A below) because we allowed the feature-specific component and eye-specific component to have different strength, controlled by w_{xF} and w_{xE}, respectively.

We did not find a noticeable advantage of the compound model over the FS model. The results of the model fit showed that the eye-specific component in stimulus-driven attention had a weight close to zero and its contribution was almost negligible (**Fig A-C** and **Table A**).

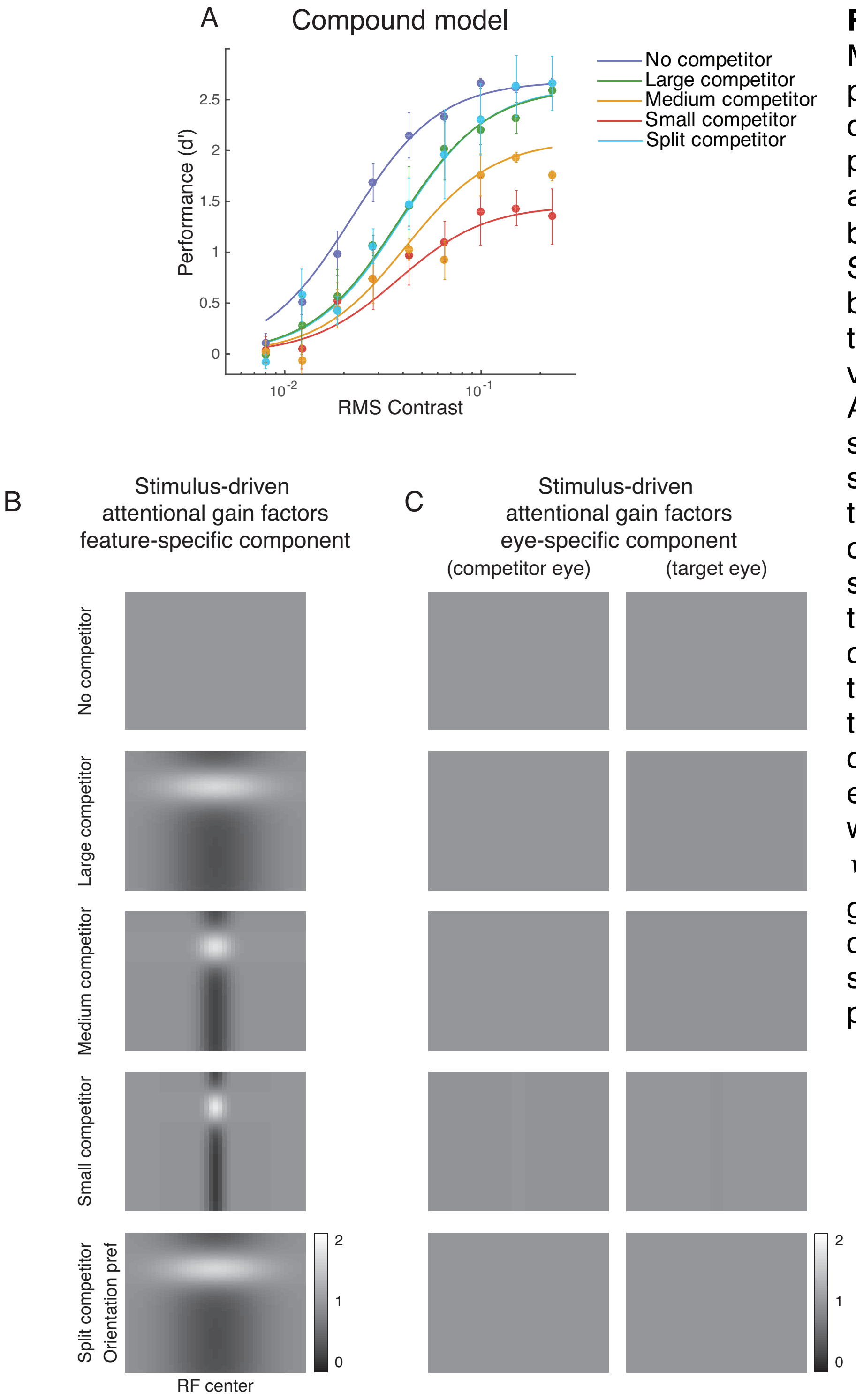

Figures A, B and C. Model fit by the compound model. A. Filled dots, psychophysical performance averaged across observers. Error bars represents ± 1 SEM. Curves are the best-fit d' by each of the two models (parameter values reported in Table A). B. The feature-specific component of stimulus-driven attention. C. The eye-specific component of the stimulus-driven attention. The eye-specific components have attentional gain factors close to uniform across all the conditions because their estimated magnitude was close to zero (see w_{xE} in Table A). The goal-driven attention components had the same form as that reported in Figure 4.

Parameter	Compound model best-fit value	Description
n	1.95	Exponent of the neural contrast response function
σ	0.0016	Constant term of the suppressive drive
w_I	0.755	Interocular normalization weight
w_{xF}	4.24<br>(1.00, 0.20, 0.26, 0.37, 0.37)	Magnitude of feature-specific component in stimulus-driven attentional modulation
w_{xE}	0.02<br>(1.00, 0.99, 0.99, 0.99, 0.99)	Magnitude of eye-specific component in stimulus-driven attentional modulation
w_v	5.03<br>(2.00)	Magnitude of goal-driven attentional modulation
p	0.14	Trade-off between the magnitude and the spatial extent of the attentional gains
σ_n	2.92	Magnitude of the noise
R^2	97.1%	

Table A. Best-fit parameter values of the compound model for the group-averaged data. The value of σ is reported in units of excitatory drive (see Equation 2 in the main text). In the rows of w_{xF} and w_{xE}, we also report the stimulus-driven attentional gain factor of the neuron tuned to the target in the no-, small-, medium-, large- and split-competitor conditions (corresponding to the five values in the parenthesis, respectively). In the row of w_v, the goal-driven attentional gain factor of the neuron tuned to the target is reported too. This value is the same across conditions because the spatial spread of goal-driven attention did not change with competitor (see details in Table 1).
