# Supplementary material for: Deconstructing Interocular Suppression: Attention and Divisive Normalization
Source: PLoS Comput Biol. 2015 Oct 30;11(10):e1004510. doi: 10.1371/journal.pcbi.1004510 (PMC4627721; doi:10.1371/journal.pcbi.1004510)
Supplement: S2 Text — (PDF) [file pcbi.1004510.s002.pdf]

Deconstructing Interocular Suppression: Attention and Divisive Normalization

Hsin-Hung Li[1], Marisa Carrasco[1,2], David J. Heeger[1,2*]

PLOS Computational Biology
(2015)

[1] Department of Psychology, New York University, New York, New York 10003
[2] Center for Neural Science, New York University, New York, New York 10003
* Corresponding author: David J. Heeger, Department of Psychology, New York University, New York, New York 10003, E-mail: david.heeger@nyu.edu

S2 Text. Model comparison by maximum likelihood estimation and Bayesian information criterion

We performed maximum likelihood estimation using group-averaged data and compared the FS and ES models using a Bayesian information criterion. We assumed that the observed group-averaged *d'* in each condition came from a normal distribution centered at the predicted *d'* by neural responses. The log-likelihood of our data can be written as:

$$l(\theta) = \log L(\theta) = \log \prod_{i=1}^{m} \frac{1}{\sqrt{2\pi}\sigma_i} \exp\left(\frac{-(d_i - q_i(\theta))^2}{2\sigma_i^2}\right)$$

$$= \sum_{i=1}^{m} \log \frac{1}{\sqrt{2\pi}\sigma_i} \exp\left(\frac{-(d_i - q_i(\theta))^2}{2\sigma_i^2}\right)$$

$$= \sum_{i=1}^{m} \log \frac{1}{\sqrt{2\pi}\sigma_i} - \frac{(d_i - q_i(\theta))^2}{2\sigma_i^2}$$

in which, θ represents the model parameters, m is the number of condition (36 conditions in Ling and Blake's data: 9 target contrast and 4 different competitors; 45 conditions in our data: 9 target contrast and 5 different competitors), d is the group-averaged *d',* and q is the *d'* predicted by the simulated neural responses in the model. Our model did not specify σ and we used the standard error of mean (of the data) in each condition as the estimate of σ when fitting the model.

Like the model fits shown in the main text, FS model and ES model had similar performance in fitting Ling and Blake's data ($\Delta BIC = 0.4$). The FS model had a smaller BIC value than the ES model when fitting our data ($\Delta BIC = 15.7$), indicating a better fit (**Fig D** and **Table B**).

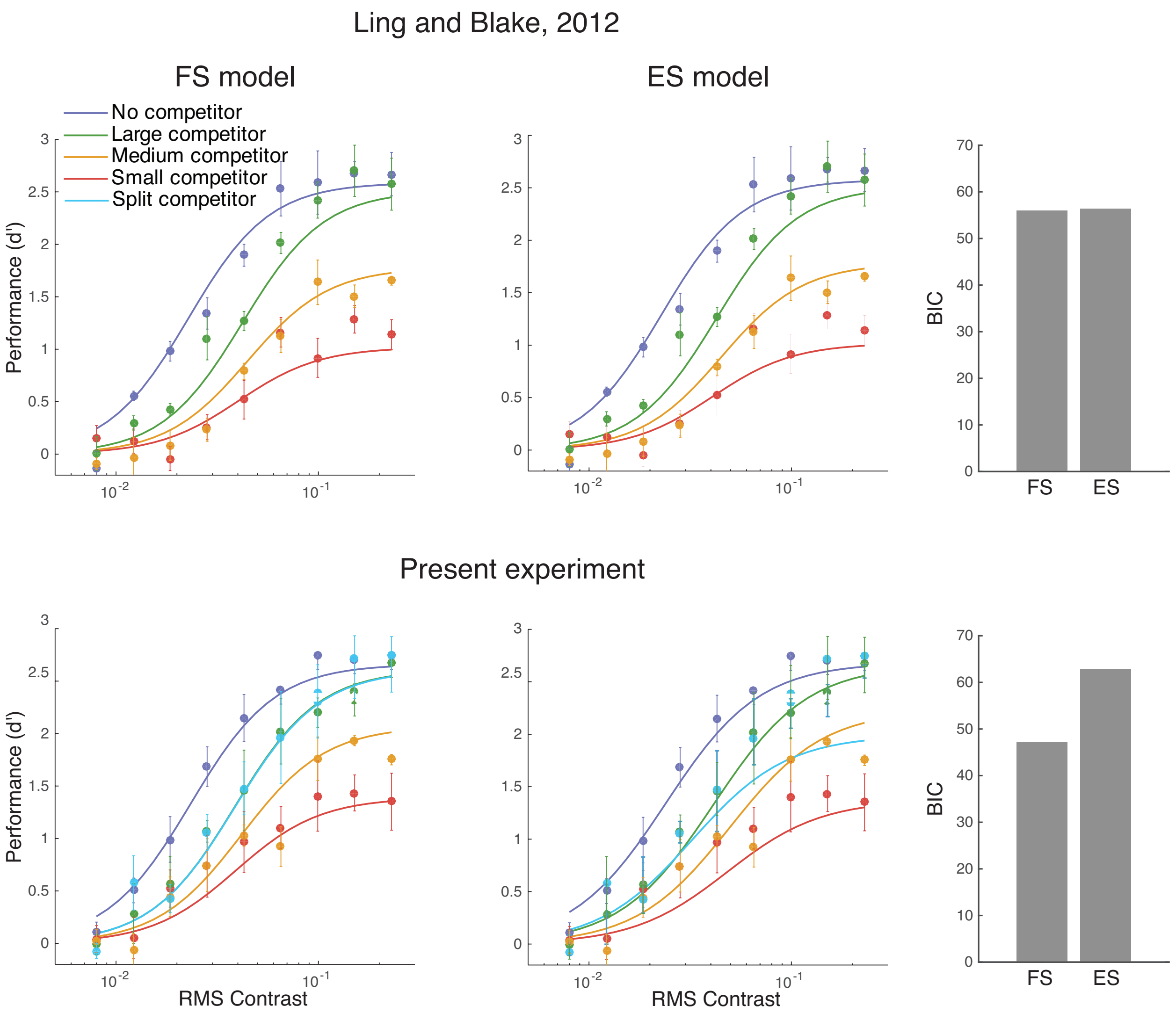

Figure D. FS and ES models fits by maximum likelihood estimation. The model fits for Ling and Blake's data (2012) are in the top row. The model fits for the present experiment are in the bottom row. Filled dots, psychophysical performance averaged across observers. Error bars represents ± 1 SEM. Curves are the best-fit d' from each of the two models (parameter values reported in **Table B**). The bar graphs in the right panels are the Bayesian information criteria for each dataset and model.

	Ling & Blake, 2012		Present data		
**Parameter**	**FS model**	**ES model**	**FS model**	**ES model**	**Description**
n	2.17	2.17	2.09	1.88	Exponent of the neural contrast response function
σ	0.0018	0.0017	0.0018	0.0018	Constant term of the suppressive drive
w_I	0.13	0.11	0.19	0.86	Interocular normalization weight
w_x	4.66	2.32	4.37	2.37	Magnitude of stimulus-driven attentional modulation
w_v	5.04	5.04	5.03	4.99	Magnitude of goal-driven attentional modulation
p	0.11	0.11	0.15	0.36	Trade-off between the magnitude and the spatial extent of the attentional gains
σ_n	3.27	3.28	3.09	2.84	Magnitude of the noise
BIC	56.0	56.4	47.2	62.9	

Table B. Best-fit parameter values from maximum likelihood estimation. The value of σ is reported in units of excitatory drive (see Equation 2 in the main text).
